# Phenotypic Spectrum of STXBP1 Gene Mutations in an Emirati Case Series

**DOI:** 10.7759/cureus.46239

**Published:** 2023-09-29

**Authors:** Nikhil Pawar, Fatima Farid Mir, Saja Tahir, Pawan Kashyape, Mohamed O E Babiker

**Affiliations:** 1 Pediatric Neurology, Al Jalila Children's Speciality Hospital, Dubai, ARE; 2 Pediatrics, Dubai Health Authority, Dubai, ARE; 3 Pediatrics, Al Jalila Children’s Speciality Hospital, Dubai, ARE

**Keywords:** uae, case series, developmental delay, seizures, stxbp1 gene mutation, genetic disease, epilepsy, neurology, pediatrics

## Abstract

Genetic mutations are increasingly recognized as etiologic factors for epilepsy and neurodevelopmental disorders. Loss of function mutations in STXBP1, one of such genes, has, in recent years, been demonstrated to cause a broad spectrum of epilepsy syndromes and chronic neurodisabilities. Syntaxin-binding protein 1 (STXBP1) is a well-recognized membrane trafficking protein responsible for synaptic transmission and is expressed ubiquitously across the brain. Our case series presents the neurodevelopmental phenotype of children with STXBP1 mutations and is the first to be reported in an Emirati patient cohort. We gathered data on five children with genetically confirmed STXBP1 mutations, each displaying varying symptomatology, EEG features, response to antiepileptic medications, and eventual disease progression. This report reveals that a majority of STXBP1 mutations were de-novo in origin; heterozygous; pathogenic to likely pathogenic variants; clinical disease onset was predominantly during infancy in the form of developmental delays with or without seizures; most of the children had co-existing ADHD or autism spectrum disorders; typical seizure semiology at onset was in the form of infantile spasms, progressing to a melange of mixed seizure types; seizure control on antiepileptic drug therapy was variable, with all cases requiring more than two medications; global developmental delay was noted in all studied children; and MRI brain findings were unremarkable in all cases. This case series demonstrates a degree of uniformity of STXBP1 mutation disease phenotypes with international literature and provides a unique insight into the genetic profile of affected children within the Emirati population.

## Introduction

Syntaxin-binding protein 1 (STXBP1) is a member of the SEC1 group of membrane trafficking proteins that interact with SNARE proteins, aid synaptic docking vesicles at presynaptic active zones, and allow neurotransmission [1]. STXBP-1 is expressed ubiquitously across the brain and neocortex both in-utero as well as post-natally [2]. There is a 3% estimated prevalence of de novo STXBP1 mutations in severe childhood epilepsy [3] and 0.25-0.5% in those with unspecified developmental disorders [4-5]. De novo STXBP1 genetic variants have been demonstrated to result in a spectrum of epilepsies such as infantile spasms, Lennox-Gastaut syndrome, West syndrome, Ohtahara syndrome, and Dravet syndrome [6]. Our case series investigates a group of five Emirati children with genetically confirmed STXBP1 mutations. This report aims to enrich the pre-existing database on STXBP1 mutations and to provide a unique insight into the genetic profiles and disease phenotypes among Emirati children. 

## Case presentation

Case one

U.A. is a seven-year-old male, firstborn to healthy, non-consanguineous parents. He was delivered at term via normal vaginal delivery with average birth weight and uneventful antenatal/post-natal periods. He has no family history of epilepsy. He achieved age-appropriate developmental milestones. He was fully immunized and fed with breast and formula milk.

The patient was well up to two months old when he first developed right-sided tonic-clonic seizures. Sodium valproate therapy was initiated in Russia at seizure onset but did not control seizures. At four months of age, the child was evaluated at our hospital. Electroencephalography (EEG) revealed atypical hypsarrhythmia, and the MRI brain was normal. Seizure freedom was achieved with the addition of vigabatrin and prednisolone therapy. He remained on tapering doses of levetiracetam until four years and stopped at six years when EEG was normal. At six years, U.A. exhibited global developmental delay (speech more than motor), truncal ataxia, and autism.

Whole-exome next-generation sequencing revealed a heterozygous likely pathogenic variant c.560C>G; p.Pro187Arg in the gene STXBP1, which was absent in both parents (de-novo mutation).

On outpatient assessment at seven years of age, he can walk without help, goes upstairs with one handheld, has truncal ataxia, can self-feed crackers, uses a spoon, says a few words like bus and mama, and has improving comprehension of spoken speech. He obeys simple commands and performs well in special needs school. On examination, growth was normal, there was no dysmorphism, and he had significant axial hypotonia with brisk deep tendon reflexes. He remained stable with ongoing physiotherapy, occupational therapy, hydrotherapy, and speech stimulation.

Case two

Z.H. is a two-year-old male, born to healthy, non-consanguineous parents and delivered at term by normal vaginal delivery with an uneventful antenatal period. He has no family history of epilepsy.

On day two after birth, he started having multifocal clonic and subsequently myoclonic seizures. Initially, there was documented hypoglycemia, but the seizures continued despite the above correction. He was treated with phenobarbitone, and later on, levetiracetam was added. A brain MRI showed no significant abnormalities apart from delayed myelination.

At three months of age, the infant continued to have daily and multiple seizures of myoclonic and multifocal clonic nature. Extensive metabolic tests were negative. An EEG done at this time showed features of hypsarrhythmia with burst suppression. With an early infantile myoclonic encephalopathy diagnosis, the child was treated with steroids, vigabatrin, pyridoxine, and levetiracetam. Seizures persisted; hence, clobazam, topiramate, and valproic acid were tried to no avail. He was admitted many times for refractory and prolonged seizures.

Seizure semiology changed by 12 months. He started having atonic seizures, brief axial tonic seizures, and absences. The features were clinically suggestive of Lennox Gastaut Syndrome (LGS). Repeated serial EEGs were classical of epileptic encephalopathy but never showed the typical EEG features of LGS. Failure of various drug combinations led to the initiation of the ketogenic diet, but this again did not significantly improve his seizures. Whole-exome next-generation sequencing revealed a novel heterozygous pathogenic mutation c.1651C>T; p.Arg551Cys in the gene STXBP1, absent in both parents (de novo). Later, the child underwent vagal nerve stimulation (VNS).

Currently, the child is on the following medications: levetiracetam, clobazam, valproic acid, and VNS. He is still getting seizures once in two days. These are now brief axial tonic seizures, lasting 1-2 minutes. In addition, he gets prolonged absences. Earlier, he used to have myoclonic-astatic seizures (improved with clobazam). There has been no developmental progression. At the age of 28 months, the child cannot sit or stand without support, has no visual following of the parents, and has no recognition of parents. He does not attempt to communicate at all, thus showing autistic tendencies. Neurologically, he has poor visual following, axial and limb hypotonia, and brisk reflexes. There is no organomegaly. He is undergoing regular physiotherapy and occupational therapy and plans to consider corpus callosotomy in a specialized center are underway.

Case three 

S.Y. is a two-month-old female, born at full term to a normal pregnancy. The parents are both healthy with no family history of epilepsy.

She was normally developing until the age of two months, when she presented with multiple daily focal seizures involving the left side of the body. Later, her seizure semiology changed to having several daily staring episodes and unresponsiveness. An EEG done at the time showed an abnormal and asymmetric background with the right hemisphere appearing slower and of higher amplitude. Interictal, bilateral multifocal epileptiform discharges were also seen. MRI was normal except for subtle variations in the posterior perisylvian cortical arrangement compared to the age. Levetiracetam followed by oxcarbazepine was started, but the seizures continued unabated. She required multiple admissions to control her seizures.

A month later, along with poor seizure control, the child had developmental regression, losing her social smile and visual fixation. A repeat EEG showed worsened multifocal discharges and a poorly organized background suggestive of epileptic encephalopathy. Treatable epileptic encephalopathies were ruled out when her seizures showed no response to pyridoxine, biotin, and leucovorin treatments. Metabolic workup tests were also normal. Lastly, whole-exome next-generation sequencing detected de-novo heterozygous pathogenic variant c.1315A> T in the STXBP1 gene. All previous medications were stopped, and the child was started on vigabatrin and a course of steroids resulting in better control of her seizures. Despite the improvement in her seizure control, she is still developmentally delayed at age six months.

Case four 

A.S. is a five-year-old male, born at full term to an uncomplicated pregnancy. He is the firstborn to first-degree consanguineous parents. His mother's family has a cognitive disability of an unknown etiology and Turner syndrome.

At six days of age, he was admitted with non-projectile vomiting, hypoactivity, and poor feeding. In addition, the mother reported an episode of peri-oral cyanosis that was resolved by stimulation. At two months of age, he presented with clusters of infantile spasms. Each cluster would last for about 10 minutes and reach 10 times a day. His symptoms were also associated with dyskinetic disorder features. The EEG was suggestive of epileptic encephalopathy with possible evolving hypsarrhythmia. An MRI brain and an initial basic metabolic workup were both normal. He was treated with phenobarbitone and topiramate. However, the child did not tolerate topiramate; hence, it was stopped, and levetiracetam was started. His frequency of infantile spasms reduced, and he showed some response to the treatment.

A month later, at a follow-up, his prolonged EEG showed hypsarrhythmia. Oral steroids were started, and phenobarbitone was tapered off. After starting steroids, the frequency of his seizures improved. At the time, whole-exome next-generation sequencing detected a heterozygous frameshift mutation c.910_911delAC [p.Thr304Profs*9] in exon 11 of the STXBP1 gene, consistent with the diagnosis of infantile epileptic encephalopathy.

Steroids were tapered off, and levetiracetam was continued. However, the semiology of the seizures changed to frequent facial twitching associated with upper extremities shaking. Clobazam was added but showed suboptimal control of his seizures. A repeat EEG showed frequent multifocal spikes and wave discharges, higher voltage than the previous one with intermittent episodes of burst suppressions. The above-mentioned anti-epileptic medications were tapered off. After initiating vigabatrin and sodium valproate, the patient has shown an excellent response to the treatment and good seizure control.

Currently, at the age of five years, he has been seizure-free since starting vigabatrin and sodium valproate at the age of two years. At the moment, he is off anti-seizure medications, and he remains seizure-free. In terms of his development, he is still delayed. He does have good head control and can roll over, but he cannot sit without support and cannot reach for or catch objects. He tracks but still cannot regard familiar faces, requiring corrective lenses. In addition, he still exhibits dyskinetic movement symptoms.

Case five 

S.S. is a 17-year-old female born at full term to an uneventful pregnancy and a normal neonatal period. She was born to consanguineous parents, with the only significant family history being epilepsy in her father's cousin.

From early on, she had significant issues with feeding and vomiting. Following that, she was noticed to be slow in acquiring her developmental milestones. She walked at the age of three years, and she never fully developed her language and speech. She uses many words, but her ability to speak in sentences is still limited. In terms of her social skills, she has significant challenges with activities of daily living and requires constant supervision. She also has hyperactive and inattentive symptoms with some autistic features.

Furthermore, she has had tremors since the age of three, primarily in both arms; this has been slowly improving. Otherwise, she never had any other associated symptoms. Both MRI brain and metabolic screening tests were reported as normal. Whole-exome next-generation sequencing showed a de novo mutation in the c.1099C>T p.(Arg367*) variant, detected in heterozygosity in the exon 13 (of 20) of the STXBP1 gene.

Table 1 summarizes the five cases presented in this report.

**Table 1:** Phenotypic features of STXBP1 mutations AEDs = antiepileptic drugs, EEG = electroencephalogram, MRI = magnetic resonance imaging, ADHD = attention deficit hyperactivity disorder

Case	Age of seizure onset	Seizure types	AEDs	EEG features	Off AEDs	Seizure control/response to therapy	Development/ outcome	Comorbidity	MRI brain findings	Diagnosis- gene mutation	Mutation nature	Zygosity	Type	Father	Mother
One	2 months	Infantile spasms	>2	Modified hypsarrhythmia	Yes	Seizure-free	Global delay	Autism	Normal	STXBP1	De-novo	Heterozygous	Likely pathogenic	Normal	Normal
Two	Neonatal	Mixed/multiple types	>2	Modified hypsarrhythmia	No	Frequent	Global delay	Autism	Normal	STXBP1	Novel	Heterozygous	Pathogenic	Normal	Normal
Three	2 months	Mixed/multiple types	>2	Modified hypsarrhythmia	No	Frequent	Global delay	None	Normal	STXBP1	De-novo	Heterozygous	Likely Pathogenic	Normal	Normal
Four	6 months	Infantile spasms	>2	Modified hypsarrhythmia	No	Seizure-free	Global delay	Autism Movement disorder	Normal	STXBP1	De-novo	Heterozygous	Pathogenic	Normal	Normal
Five	No seizures	No seizures	Not required	Not performed	Not applicable	Not applicable	Global delay	Autism ADHD Movement disorder	Normal	STXBP1	De-novo	Heterozygous	Pathogenic	Normal	Normal

## Discussion

Early Infantile Developmental & Epileptic Encephalopathy (EIDEE) was previously known as early myoclonic encephalopathy (EME) and early infantile epileptic encephalopathy (EIEE). Common etiologies include structural malformations, metabolic disorders, and underlying genetic defects [7]. STXBP1 gene mutations represent a relatively common cause of epileptic encephalopathy, and the diagnosis remains challenging as phenotypic characteristics are still under study.

Literature to date has shown consistency in the following disease patterns: majority with onset in infancy, severe global adaptive impairments, hyperactivity, receptive and social impairment, the spectrum of seizure types, and presence of ataxia/dyskinesia [2,8-12]. The most commonly observed EEG abnormality is focal/multifocal epileptic activity, followed by a burst suppression pattern and then hypsarrhythmia [6]. MRI brain is usually normal, but cases with cortical thinning and hypo-myelination have also been described [6]. Neurodevelopmental outcomes are generally poor with significant intellectual disability and developmental delays.

Our series of five patients have exhibited onset age within infancy, typically beginning with infantile spasms. A minimum of dual anti-epileptic therapy was required, and all except one patient achieved seizure freedom. Electroencephalography in all cases demonstrated a pattern of hypsarrhythmia. With time, disease phenotype in all children had broadened to include tonic-clonic, multifocal, and myoclonic seizures. Two patients also developed a non-epileptic movement disorder. Intellectual abilities were severely impaired in all of them. Behavior problems, such as autism and physical examination features of axial hypotonia, with brisk deep tendon reflexes were manifest in all our patients. STXBP1 mutations had arisen de-novo in four of our cases.

## Conclusions

The presented case series echoes the knowledge that STXBP1 gene mutations have a spectrum that includes various epilepsy syndromes, autistic features, movement disorders, and degrees of neurodevelopmental impairment. The age at presentation is classically within infancy. Onset is typical with infantile spasms, which require more than one anti-epileptic drug to achieve control. Global developmental delay and co-existent autistic features are almost uniformly present and most pathogenic STXBP1 gene mutations arise de-novo.
